# Copula-Based Assessment and Regionalization of Drought Risk in China

**DOI:** 10.3390/ijerph20054074

**Published:** 2023-02-24

**Authors:** Ming Li, Guiwen Wang, Shengwei Zong, Xurong Chai

**Affiliations:** 1 College of Geographical Sciences, Shanxi Normal University, Taiyuan 030031, China; 2 Key Laboratory of Geographical Processes and Ecological Security of Changbai Mountains, Ministry of Education, School of Geographical Sciences, Northeast Normal University, Changchun 130024, China; 3 School of Geographical Sciences, Northeast Normal University, Changchun 130024, China

**Keywords:** meteorological drought, run theory, copula function, drought risk

## Abstract

Droughts are widespread in China and have brought considerable losses to the economy and society. Droughts are intricate, stochastic processes with multi-attributes (e.g., duration, severity, intensity, and return period). However, most drought assessments tend to focus on univariate drought characteristics, which are inadequate to describe the intrinsic characteristics of droughts due to the existence of correlations between drought attributes. In this study, we employed the standardized precipitation index to identify drought events using China’s monthly gridded precipitation dataset from 1961 to 2020. Univariate and copula-based bivariate methods were then used to examine drought duration and severity on 3-, 6-, and 12-month time scales. Finally, we used the hierarchical cluster method to identify drought-prone regions in mainland China at various return periods. Results revealed that time scale played an essential role in the spatial heterogeneity of drought behaviors, such as average characteristics, joint probability, and risk regionalization. The main findings were as follows: (1) 3- and 6-month time scales yielded comparable regional drought features, but not 12-month time scales; (2) higher drought severity was associated with longer drought duration; (3) drought risk was higher in the northern Xinjiang, western Qinghai, southern Tibet, southwest China, and the middle and lower reaches of the Yangtze River, and lower in the southeastern coastal areas of China, the Changbai Mountains, and the Greater Khingan Mountains; (4) mainland China was divided into six subregions according to joint probabilities of drought duration and severity. Our study is expected to contribute to better drought risk assessment in mainland China.

## 1. Introduction

Drought is one of the most severe climate-related threats to human civilization, with randomness, creep, and complexity [1,2]. It refers to a temporary lack of precipitation or water shortage in a specific period, irrespective of the region’s typical aridity. Consequently, droughts can take place in both wet and dry regions [3,4]. Drought can generally be defined from various aspects such as meteorology, hydrology, agriculture, and socio-economics. Currently, droughts are commonly monitored and analyzed through various drought indices obtained from gauge-based or remote-sensing data. The widely-used climate-related drought indices include the Palmer Drought Severity Index (PDSI) [5], Standardized Precipitation Index (SPI) [6], Standardized Precipitation Evaporation Index (SPEI) [7], and so on. Our research is limited to meteorological drought as evaluated by SPI, which has been proven to be an effective index for assessing drought severity and duration from regional to global scales [8,9,10,11,12]. The World Meteorological Organization and a few international meteorological drought centers have also suggested the SPI as one reference drought index to track drought features, due to its easy computation, multi-time scale properties, and spatiotemporal comparability [13,14].

Drought events are intricate, stochastic processes with multi-attributes (e.g., duration, severity, intensity, and return period). Hence, probabilistic and stochastic methods are commonly used to explore various drought features. The run theory, introduced by Yevjevich [15], has been widely adopted in drought analysis, as confirmed by a large number of studies [16,17,18]. Prior to 2006, univariate analysis was the most popular method employed to investigate drought frequencies and return periods, involving drought duration, severity, maximum severity, etc. [19,20]. However, as a result of the correlations between the various drought attributes, univariate analysis cannot fully and objectively describe the intrinsic features of droughts. As such, some researchers have extended their analysis from univariate to bivariate analysis to gain a deeper understanding of drought occurrences [21,22]. Nonetheless, this approach assumes that all drought attributes follow the same marginal distribution, which may not always be practical.

Copula functions make it possible to describe the dependence structure of drought multi-attributes, without the restrictions of the marginal distributions. This is particularly advantageous because it provides a comprehensive understanding of how different drought variables relate to each other, and how their joint behavior influences the overall drought risk. By exploring the copula-based relationships between drought variables, researchers can identify hidden patterns, causal factors, and key drivers of drought, which would be difficult to uncover using traditional statistical methods. Scholars [8,23,24,25] have extensively used this method to analyze the frequency and return period of droughts since its first application in meteorological drought by Shiau [26]. However, most studies focused on the properties of copula functions or the joint distribution of drought variables at specific meteorological stations or small areas. It should be noted that one drought event generally covers a large area, and so do the drought impacts. Hence, the drought frequencies derived from discrete stations cannot well depict drought distributions and regional characteristics in continuous space. However, several studies have demonstrated that gridded data can compensate for the defects of uneven or sparse distributions of meteorological observation stations [17,27,28].

In the context of global warming, droughts in China have expanded in terms of extent, severity, and frequency [29,30,31,32,33]. Numerous studies have been conducted on the spatiotemporal characteristics of drought across the country, leading to many noteworthy accomplishments. For instance, a severe drought in 1997 led to the lower reach of the Yellow River experiencing 226 days with zero flow [31]. Southwest China, in the period of summer 2009 and spring 2010, was hit by a once-in-a-century drought, which caused water shortages for over 16 million people and 11 million livestock [32]. In 2011, China experienced a widespread and severe drought event that affected regions across the country [34]. Notably, the Yangtze River Basin, a typically humid region, suffered the worst drought in 50 years in the spring of 2011. Han et al. [33] revealed a broadening of the drought-affected region in China, accompanied by an escalation in both the frequency and severity of drought events. Nonetheless, previous studies on droughts in mainland China have not explored the probabilistic behavior and frequency-magnitude relationships of drought characteristics using copula-based bivariate frequency analysis. Additionally, no attempt has been made to investigate the spatial patterns of drought risk indicators to identify regions that are prone to droughts.

This study aims to comprehensively examine the spatial characteristics of drought events in mainland China over the past 60 years, from both univariate and bivariate perspectives. The objectives are four-fold: (1) to identify and characterize drought events and associated variables using run theory; (2) to analyze the spatial patterns of various drought characteristics, including duration, severity, maximum severity, and frequency; (3) to perform traditional univariate and copula-based bivariate frequency analyses on drought duration and severity; and (4) to conduct drought risk regionalization.

## 2. Materials and Methods

### 2.1. Data Source and Collection

**Figure 1:**
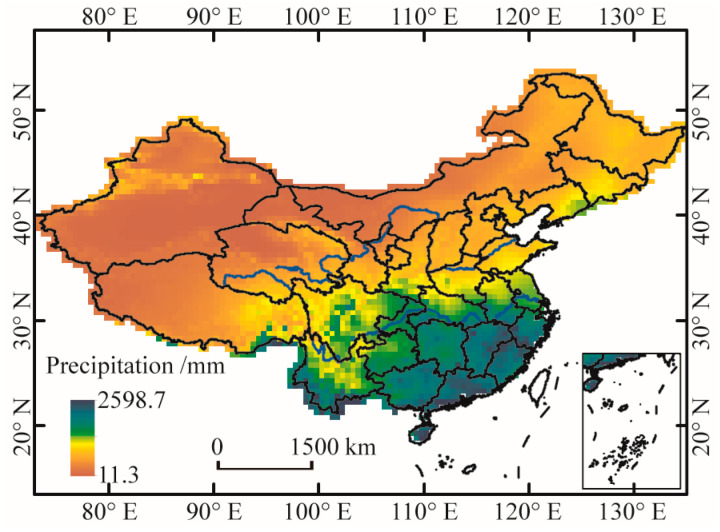
Spatial pattern of average annual precipitation for the period 1961–2020.

In this study, the utilized monthly gridded precipitation dataset (version 2.0) has a spatial resolution of 0.5° × 0.5° and encompasses a time span ranging from 1961 to 2020. The dataset was acquired from the China Meteorological Data Service Centre [35]. Thin-plate smooth spline interpolation was employed to construct the gridded dataset based on the precipitation observations from 2472 meteorological stations across China [36]. Excluding Taiwan and South China Sea Islands, the dataset comprises 4189 grids and was verified by average root-mean-square and cross-validated by the Tropical Rainfall Measurement Mission product [36,37]. As a result, the dataset has an average error of 0.49 mm/month, and the average correlation coefficient between the grids and the observations is 0.93 (arriving at a significance level of α = 0.01) [38]. The dataset has also been extensively applied for research on climate change in China [1,30,39]. The multi-year average precipitation was calculated using this dataset and is shown in Figure 1. The spatial pattern of annual precipitation indicates a gradual decrease from the southeastern coast to the northwest inland. In addition, the seasonal distribution of precipitation is also uneven, which mainly falls in May-October in the form of high-intensity rainforms [40,41]. The uneven spatial distribution of precipitation and the considerable disparities in the time domain have led to frequent droughts and floods in China, which have greatly threatened agricultural production, as well as people’s lives and properties.

### 2.2. Methods

#### 2.2.1. Standardized Precipitation Index

The SPI index proposed by Mckee et al. [6] can characterize droughts on various time scales, such as 1-, 3-, 6-, and 12-month periods. The detailed computation formulas can be found in the previous studies [10,42,43], and the key steps are as follows [44,45]:

(1) Calculating the cumulative precipitation on a specific time scale;

(2) Matching the probability distribution of accumulative precipitation using a gamma distribution function;

(3) Determining the non-exceedance probabilities for cumulative precipitation;

(4) Converting the probabilities to a standard normally distributed variable.

SPI is classified into five groups based on the “Meteorological drought grade (GB/T20481-2017)” [46], as shown in Table 1.

SPI values over different time scales can depict drought types [27]. In this study, we employed SPI on the time scales of 3-month (SPI-3), 6-month (SPI-6), and 12-month (SPI-12) to explore the short-, medium-, and long-term drought characteristics in mainland China (excluding Taiwan and South China Sea Islands). The SPI value of a specific accumulation period was assigned to the final month of that period, so the SPI-6 value for June 2020 referred to the SPI for the accumulative precipitation from January to June 2020.

#### 2.2.2. Run Theory

**Figure 2:**
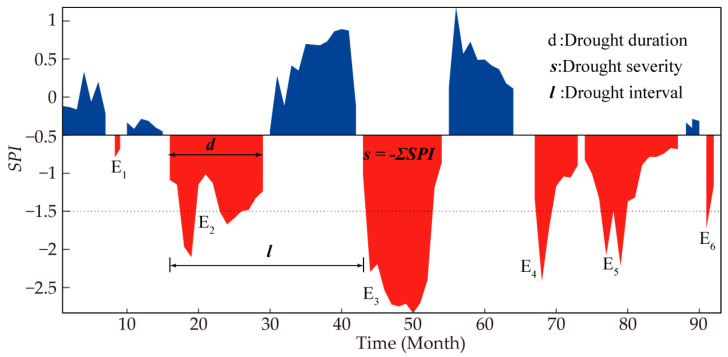
A basic illustration of drought events based on run theory. The red portions indicate drought periods, while the blue portions indicate non-drought periods.

The run theory introduced by Yevjevich [15] makes it easy to identify drought features including start and end times, as well as the duration and severity of drought. For this study, a drought threshold of −0.5 was set based on Table 1. There were two aspects to be dealt with in the process of drought identification: (1) Minor drought events. When SPI was less than −0.5, we preliminarily judged that the month was one drought-month. It can be seen in Figure 2 that there were six distinct droughts labelled E_1_, E_2_, E_3_, E_4_, E_5_, and E_6_. A single-month drought event was considered to have occurred if its associated SPI value was less than −1.5 (such as E_6_). Otherwise, it was considered a minor drought event and should be ignored (such as E_1_); (2) A confluence of droughts. In the case of two consecutive droughts separated by a month, if the SPI value was between −0.5 and 0.5 in the interval month, the two adjacent droughts were considered subordinate droughts. Then, the two droughts should be combined into one drought event (such as E_4_ and E_5_). The total drought duration equaled E_4_(*d*) + E_5_(*d*) + 1, while the drought severity equaled E_4_(*s*) + E_5_(*s*). Otherwise, it was two independent drought events. As a result, four drought events were finally identified, namely E_2_, E_3_, E_4_ + E_5_ and E_6_. Wherein drought duration (*d*) refers to the number of months elapsed between its onset and its cessation, while drought interval (*l*) is the amount of time between its onset and that of the next adjacent drought. The drought severity (*s*) is the cumulative difference that the SPI falls below −0.5 for each drought episode. To facilitate computation, we doubled the drought severity by −1 to get a positive value [47,48], namely:

(1) $$s=-\sum_{i=1}^{d}SPI_{i}$$

This paper discussed the drought duration, severity, and maximum (the peak value of drought severity) [49] as defining features of drought episodes. To calculate these attributes for each grid, the following formulas were used:

(2) $$s_{\mathrm{ave}}=\sum_{i=1}^{n}s_{i}/n$$

(3) $$d_{\mathrm{ave}}=\sum_{i=1}^{n}d_{i}/n$$

(4) $$s_{\max}=\underset{1\leq i\leq n}{\max s_{i}}$$

where *n* represents the total number of droughts.

#### 2.2.3. Copula Function

Sklar [50] pointed out that copula functions could show how drought variables are related even if they don’t have the same marginal distribution. They can depict the effects of drought on a specific region with more precision than conventional statistical methods.

The Archimedean copula function family, which includes Clayton, Gumbel, and Frank copulas, has been widely used in hydrology and meteorology. Selecting the appropriate copula function directly impacts the analysis and computation outcomes [51]. Thus, it is crucial to identify the copula function that can effectively represent the variables in the analysis. Here, the best-fit copula was selected based on the Akaike Information Criterion (AIC) [52]. The selection process followed a criterion where a lower value indicated better performance. The formula for AIC was provided in Equation (5). After selecting the appropriate copula function, its parameters were evaluated by the marginal inference function, which consisted of two separate estimation steps: first estimating each univariate marginal distribution function based on the maximum likelihood method; and then estimating the copula dependence parameter [53]. It should be also noted that the presence of a correlation between variables is essential in the creation of a bivariate distribution using a copula function. Without this correlation, the use of a copula function is not possible [54]. To determine the correlation between drought severity and duration in this study, Kendall’s correlation coefficient [55] was employed. Prior research [24,56] revealed that the cumulative distribution functions of drought duration (*F_D_*(*d*)) and severity (*F_S_*(*s*)) follow the exponential and gamma distributions, as defined in Equations (6) and (7), respectively.

(5) $$\mathrm{AIC}=2(k-\ln L_{\max})$$

(6) $$F_{D}(d)=1-e^{-d/\lambda },\;\;\;\;\;\;d>0$$

(7) $$F_{S}(s)=\int_{0}^{s}\frac{s^{\alpha -1}}{\beta^{\alpha }\Gamma (\alpha )}e^{-s/\beta }ds,\;\;\;\;\;\;s>0$$

where the variable *L*_max_ represents the maximum log-likelihood that can be attained by the model for a given dataset; *k* refers to the number of parameters that can be freely estimated for the model. *λ* denotes the parameter to describe exponential distribution, *α* and *β* determine the shape and scale of gamma distribution. The copula-based joint cumulative distribution function of drought duration and severity may be represented as follows:

(8) $$F_{DS}\left(d,s\right)={\left(F_{D}^{-\theta }(d)+F_{S}^{-\theta }(s)-1\right)}^{-1/\theta },\;\;\;\;\;\;\theta \geq 0$$

where *θ* is a measure for quantifying the strength of the link between *F_D_*(*d*) and *F_S_*(*s*).

#### 2.2.4. Return Period and Joint Probability

The return period of a drought refers to an estimated average time between two drought events with a specified value or higher [20]. Given that droughts may persist longer than one year, Shiau and Shen [57] developed the following formula to calculate the return period of drought duration (*R_D_*) and drought severity (*R_S_*):

(9) $$R_{D}=E\left(l\right)/\left(1-F_{D}\left(d\right)\right)$$

(10) $$R_{S}=E\left(l\right)/\left(1-F_{S}\left(s\right)\right)$$

where *E*(*l*) denotes the expected value of drought interval time as described in Section 2.2.2.

Drought risk assessment and drought management rely heavily on information on the joint probability of drought features. In this study, we assessed the joint probability that both drought duration and severity concurrently surpass the specified threshold (i.e., *D* ≥ *d* and *S* ≥ *s*). The formula for the probability was given by Shiau [23]:

(11) $$P=P(D\geq d\cap S\geq s)=1-F_{D}\left(d\right)-F_{S}\left(s\right)+F_{DS}\left(d,s\right)$$

#### 2.2.5. Drought Risk Regionalization

Drought risk regionalization is a vital task in the field of drought risk management, as it is the process of identifying areas that are likely to be affected by drought hazards. This is achieved through the aggregation of regions that have similar climatic characteristics and spatial continuity. The process of drought risk regionalization is complex, as it involves the use of sophisticated statistical and geographical techniques, such as clustering and spatial analysis. By implementing these methods, researchers can effectively identify areas that are susceptible to drought and subsequently develop strategies to mitigate its impact. In this study, we employed the HiClimR package [58] in R software to accomplish the drought risk regionalization in mainland China. The scheme for drought risk regionalization can be summarized as follows:

(1) Extract the longitude and latitude of each grid point and generate the corresponding raster layer.
(2) Select the dataset of input data for the implementation of hierarchical clustering. The dataset includes latitude, longitude, and grid-based joint probability of drought duration and severity calculated by Equation (11).
(3) Preprocess the input data by removing the effect of magnitude through standard deviation normalization.
(4) Identify adjacent grids by using the latitude and longitude variables.
(5) Calculate the distance between the grids using the Pearson correlation coefficient and the distance between classes using the sum of squares method.
(6) Determine the adjacency and homogeneity of the regions by minimizing the inter-regional correlation coefficient and maximizing the intra-regional correlation coefficient.
(7) Obtain the final group numbers based on the silhouette width criterion.
(8) Group the adjacent grids based on their similarity in terms of joint probability of drought duration and severity.
(9) Evaluate the results of the drought risk regionalization and identify the areas that are susceptible to drought hazards.

## 3. Results

### 3.1. Regional Average Drought Attributes

**Figure 3:**
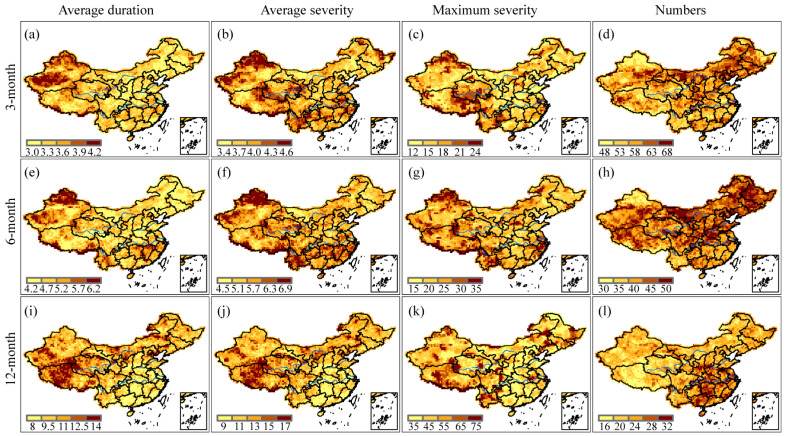
Spatial patterns of average duration, average severity, maximum severity, and the number of drought episodes derived from SPI-3, SPI-6, and SPI-12 values, respectively. Specifically, (**a**,**e**,**i**) indicate the average drought duration; (**b**,**f**,**j**) represent the average drought severity; (**c**,**g**,**k**) refer to the maximum drought severity; (**d**,**h**,**l**) denote the total number of drought events.

Drought attributes, such as average duration and severity, maximum severity, and the number of drought episodes, were depicted spatially in Figure 3 at 3-, 6-, and 12-month time scales. On the 3-month time scale, most of Xinjiang, northern and southern Tibet, western Qinghai, and eastern Heilongjiang experienced relatively longer drought durations, while the average drought durations were relatively short in eastern Inner Mongolia, southwestern Heilongjiang, eastern Jilin, and most of Shaanxi province (Figure 3a). The distribution patterns of average drought severity showed that western and northern Xinjiang, northern Tibet, southwestern and eastern Qinghai, central and eastern Yunnan, southern Yangtze River Basin, central Anhui, and eastern Heilongjiang experienced relatively more severe droughts. In contrast, the values of drought severity were relatively low in southeastern Xinjiang, western Tibet, western Inner Mongolia, and eastern Jilin (Figure 3b). Northern Xinjiang, northeastern Tibet, southern Qinghai, and central-northern Yunnan have experienced more intense droughts (Figure 3c). Compared with southern China, more droughts occurred in northern China (Figure 3d).

The average duration and severity followed similar spatial patterns on the 6-month time scale as they did on the 3-month time scale (Figure 3e,f). However, the most severe droughts were also observed along the southeastern coast (Figure 3g), and the drought frequency pattern was more pronounced, with more droughts occurring north of the Yangtze River than south of it (Figure 3h).

On the 12-month time scale, the average drought durations in Northeast China, Northwest China, North China, and Qinghai-Tibet Plateau were longer than that in the southeast coastal provinces (Figure 3i). Drought severity followed a similar pattern as drought duration (Figure 3j), which meant that longer drought duration tended to result in more severe drought. The most severe droughts occurred in southwestern China and at the junction of Inner Mongolia, Jilin, and Liaoning provinces (Figure 3k). The pattern of drought frequency was quite different from that on the 3- and 6-month time scales. Although the drought durations in central and eastern China were shorter, the drought frequencies were relatively high (Figure 3l). Similar to the drought severity, the areas with higher numbers of drought episodes decreased as time scales increased. Compared to the spatial patterns of short- and medium-term drought attributes, long-term drought attributes had quite distinct regional distributions. So, when examining drought-related issues, an appropriate time scale should be selected depending on the purpose of the study.

### 3.2. Spatial Patterns of Drought Severity and Duration with Various Return Periods

**Figure 4:**
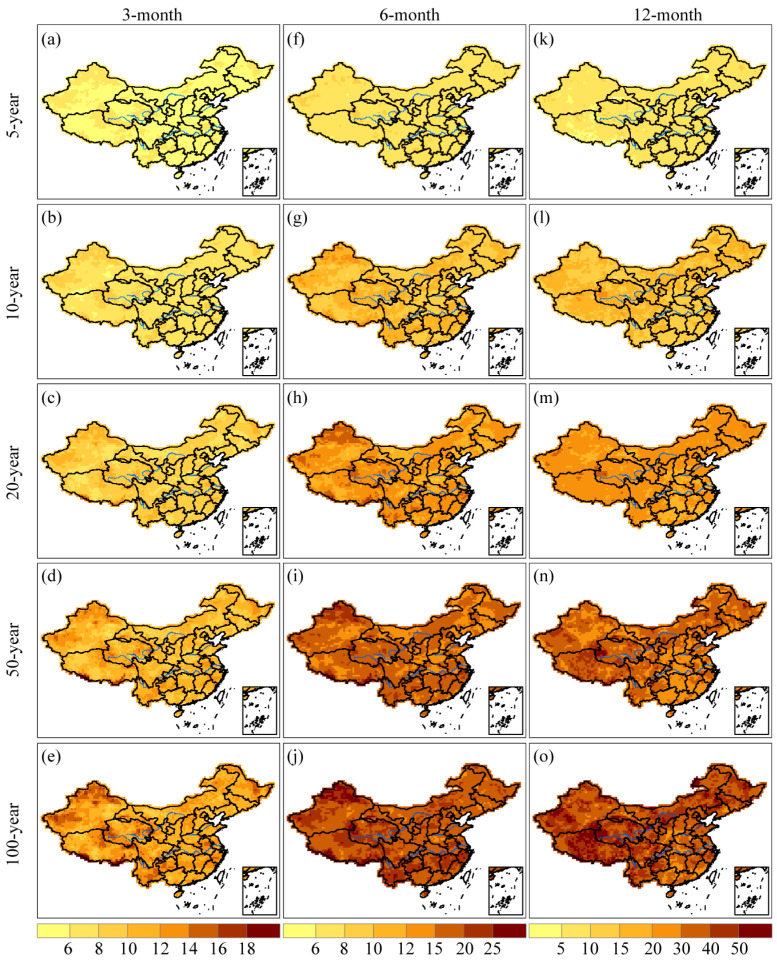
Spatial distributions of drought severity with 5-, 10-, 20-, 50-, and 100-year return periods based on SPI-3, SPI-6, and SPI-12 values. Specifically, (**a**–**e**) in each panel show the spatial patterns of drought severity with return periods of 5-, 10-, 20-, 50-, and 100-year at the 3-month time scale; (**f**–**j**) in each panel depict the corresponding patterns at the 6-month time scale; and (**k**–**o**) in each panel illustrate the patterns at the 12-month time scale.

Figure 4 illustrated the drought severity for 3-, 6-, and 12-month time scales at different return periods, including 5-, 10-, 20-, 50-, and 100-year. The study findings suggested that drought severity increased with longer drought return periods across various time scales [27]. On the 3-month time scale, severe droughts were observed in most regions of China except the Changbai Mountains, Greater Khingan Mountains, Pearl River Basin, western Tibet, and eastern Xinjiang province. Moreover, similar spatial patterns of drought severity were observed at 3- and 6-month time scales. It should be noted that severe droughts have also been found along the southeastern coast of China. On the 12-month time scale, the Qinghai-Tibet Plateau was identified to be more susceptible to droughts.

**Figure 5:**
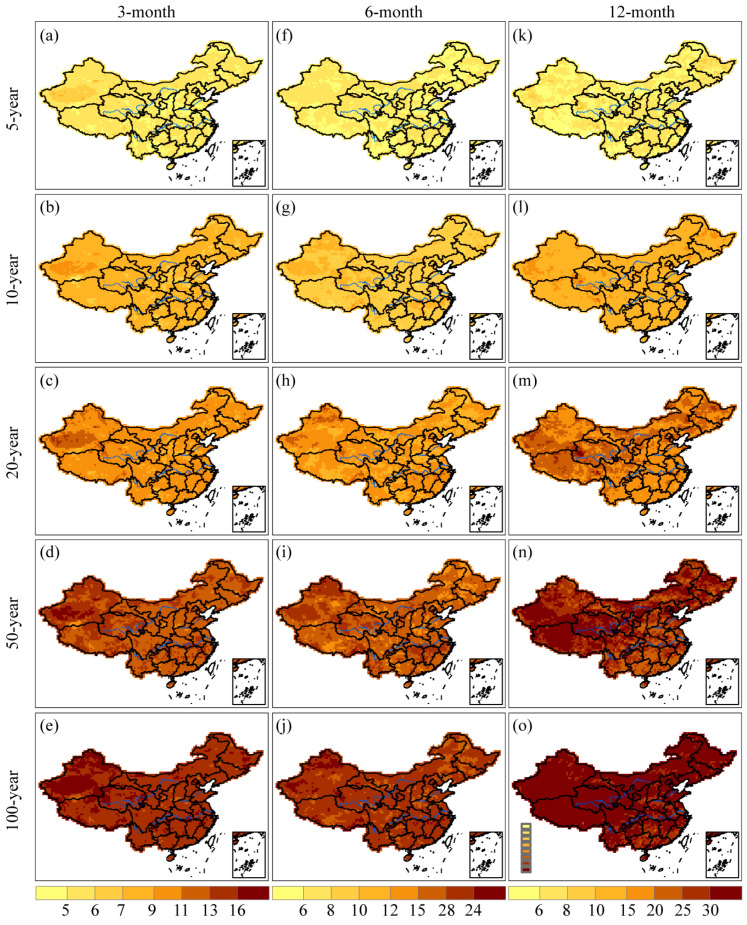
Spatial distributions of drought duration with 5-, 10-, 20-, 50-, and 100-year return periods based on SPI-3, SPI-6, and SPI-12 values. Specifically, (**a**–**e**) in each panel show the spatial patterns of drought duration with return periods of 5-, 10-, 20-, 50-, and 100-year at the 3-month time scale; (**f**–**j**) in each panel depict the corresponding patterns at the 6-month time scale; and (**k**–**o**) in each panel illustrate the patterns at the 12-month time scale.

**Figure 6:**
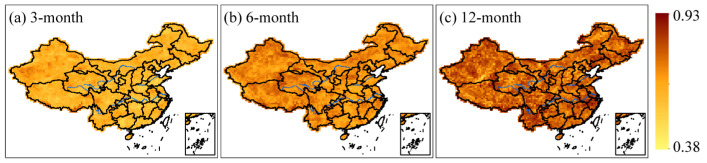
The spatial distribution of the Kendall correlation coefficients between the drought duration and severity at different time scales: (**a**) 3-month, (**b**) 6-month, and (**c**) 12-month.

Drought duration for 3-, 6-, and 12-month time scales with return periods of 5-, 10-, 20-, 50-, and 100-year were depicted in Figure 5. In comparison to Figure 4, we discovered that areas where droughts lasted longer were likewise associated with more severe droughts. This means that longer drought durations tended to indicate more severe droughts and vice versa. From Figure 4 and Figure 5, it can be observed that more severe and longer drought events were detected in western and northern Xinjiang, southwestern China, and the southern regions of the mid-lower reaches of the Yangtze River. In contrast, less intense and shorter drought events were monitored mainly in the Greater Khingan Mountains, Changbai Mountains, and Pearl River Basin. To confirm the positive relationship between drought duration and severity, we analyzed the spatial distribution of Kendall correlation coefficients for these two variables at different time scales. Specifically, we plotted the correlation coefficients for the 3-, 6-, and 12-month time scales in Figure 6. The findings indicate that there is indeed a positive correlation between drought duration and severity, and this relationship reaches a significance level of α = 0.05. Moreover, the Kendall correlation coefficient shows an increase with longer time scales, suggesting that the longer the drought lasts, the more severe it becomes. Nonetheless, univariate analysis is insufficient to completely characterize drought episodes due to the correlation between drought attributes.

### 3.3. Joint Probability of Drought Duration and Severity

**Figure 7:**
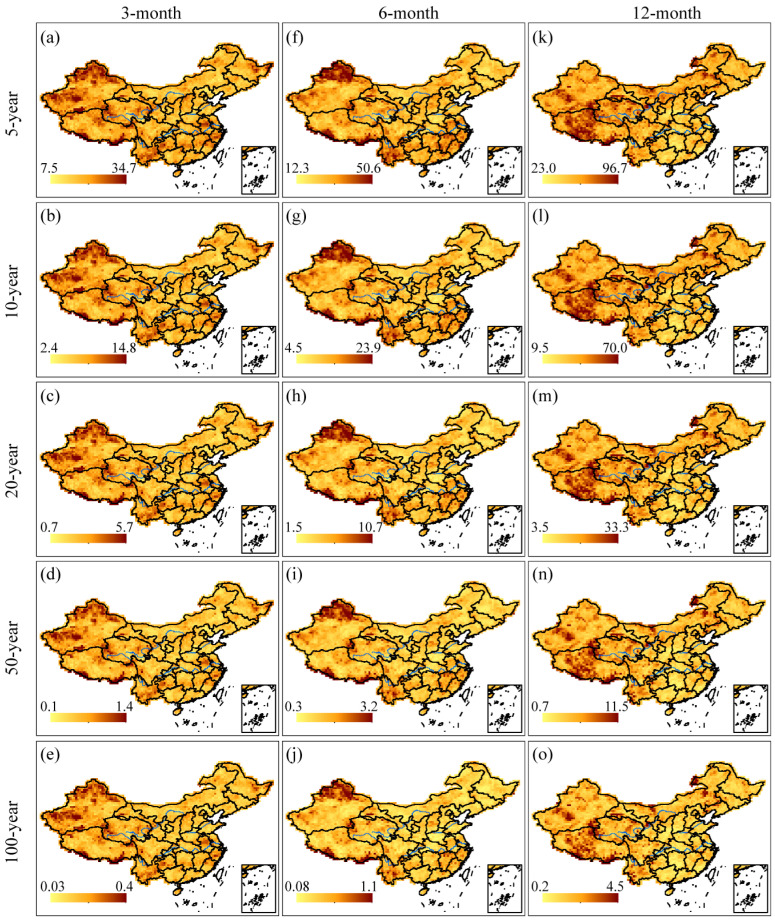
Joint probability (%) of drought duration and severity. The thresholds *s* and *d* are derived from drought severity and duration for 5-, 10-, 20-, 50-, and 100-year return periods, respectively. Specifically, (**a**–**e**) in each panel show the spatial patterns of joint probability with return periods of 5-, 10-, 20-, 50-, and 100-year at the 3-month time scale; (**f**–**j**) in each panel depict the corresponding patterns at the 6-month time scale; and (**k**–**o**) in each panel illustrate the patterns at the 12-month time scale.

To gain a better understanding of the relationship between drought severity and duration, it is essential to evaluate the drought risks in mainland China using bivariate analysis. Firstly, the best-fit copula function was determined grid-by-grid based on the AIC criteria, and the Frank copula was found to have the best performance for each grid at various time scales. Next, we employed the joint probability, as described in Equation (11), with the thresholds *s* and *d* derived from the preceding univariate study of drought severity and duration for 5-, 10-, 20-, 50-, and 100-year return periods, respectively. The results displayed in Figure 7 showed a clear spatial distribution of the joint probability *P* at various time scales and return periods. On the 3- and 6-month time scales, relatively large *P* values were mainly observed in northern Xinjiang, western Qinghai, southern Tibet, central Yunnan, and eastern Heilongjiang, indicating a higher likelihood of experiencing droughts in these regions. Conversely, the *P* values in the southeastern coastal areas of China, Changbai Mountains, and Greater Khingan Mountains were mostly smaller, implying a lower drought risk in these regions. On the 12-month time scale, the drought risk on the Tibetan Plateau was the highest, which was consistent with the results obtained from the univariate analysis.

### 3.4. Drought Risk Regionalization

**Figure 8:**
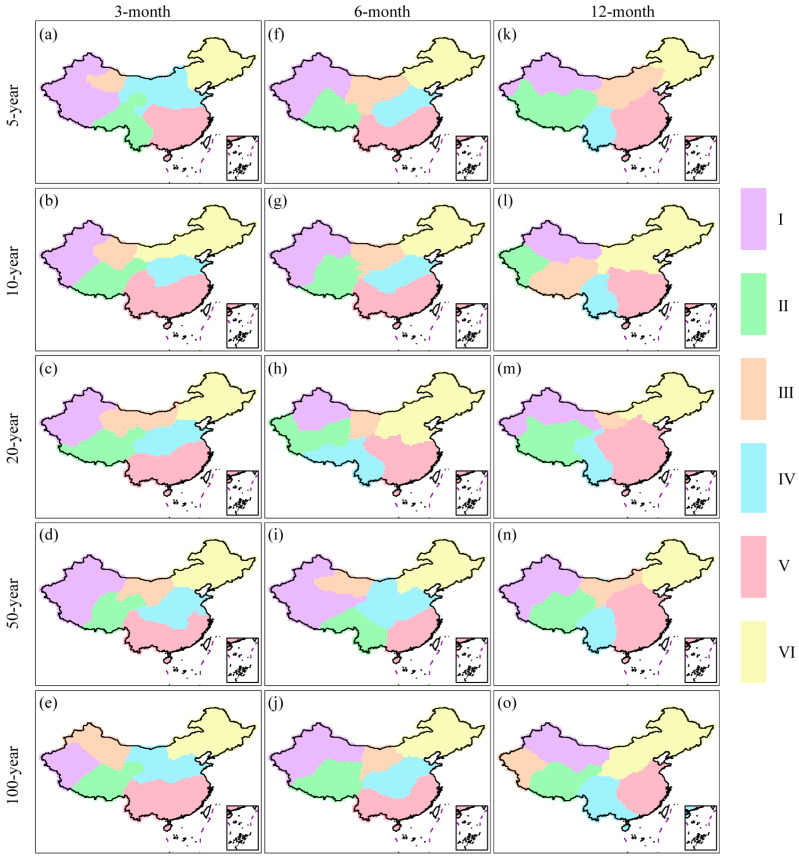
Drought risk regionalization across various return periods and time scales. Specifically, (**a**–**e**) in each panel show the patterns of drought risk regionalization with return periods of 5-, 10-, 20-, 50-, and 100-year at the 3-month time scale; (**f**–**j**) in each panel depict the corresponding patterns at the 6-month time scale; and (**k**–**o**) in each panel illustrate the patterns at the 12-month time scale.

In order to identify drought-prone regions in mainland China, we mapped the drought risk regionalization at various return periods, as shown in Figure 8. With the aid of the silhouette width criterion, a preliminary estimate of the optimal number of clusters was made. According to the findings, the optimal number of drought risk zones ranged from four to six categories over various return periods. For convenience of analysis, we divided mainland China into six sub-regions considering the topography, drought risk, and the continuity and independence of regions. The map visualization was accomplished using Arcmap 10.4. The mapping process involved the use of the focal statistics function for raster smoothing, Chinese border extraction, raster to polygon conversion, and modifying isolated classification grid points by changing their attributes to that of the adjacent major class. It was clear from Figure 8 that the spatial pattern of drought risk zones exhibited a high degree of similarity on 3- and 6-month time scales with return periods ranging from 5 to 100 years. The six sub-regions were identified as Xinjiang (I), Qinghai-Tibet Plateau (II), central and western Inner Mongolia (III), South China (IV), North China (V), and Northeast China (VI). However, for the 12-month time scale, the zoning results under different return periods mostly separated Southwest China, suggesting that this region was particularly susceptible to long-term droughts. In addition, the zone (central and western Inner Mongolia) was absorbed by neighboring zones.

## 4. Discussion

### 4.1. Drought Characteristics and Risk

The study observed that regions with less precipitation and high rainfall variabilities, such as Northwest China and the Qinghai-Tibet alpine region, experienced longer and more severe droughts. Several studies have highlighted that the water resource supply is decreasing, which makes these regions more susceptible to drought [59,60,61,62]. However, other studies have shown an increase in precipitation in the Qinghai-Tibet Plateau and Northwest China, indicating a possible improvement in drought conditions in these regions in the future [63,64]. Additionally, severe drought episodes were observed in the middle and lower reaches of the Yangtze River and the Yunan-Guizhou Plateau, which might have been due to a significant reduction in precipitation [12,16,17,65]. Another study conducted by He et al. [66] identified a higher drought risk in the northern Xinjiang, Loess Plateau, Northeast Plain, southern areas of the Yangtze Plain, and Yunnan-Guizhou Plateau. Our research, as depicted in Figure 7, also reveals similar findings, although we identified high drought risk in eastern Heilongjiang instead of most of the Northeast Plain. The reason for the discrepancy might be that our primary focus was meteorological drought rather than agricultural drought, which was the main concern in their study.

### 4.2. Regionalization

In China, previous studies on regionalization focused on employing various indices to determine climate zones. For example, Yang and Li [67] applied the rotated empirical orthogonal function (REOF) method to divide China into 11 subregions based on the precipitation anomaly percentage. However, due to the empirical nature of the REOF method, there is a certain subjectivity and fuzziness in determining the boundaries of the subregions. Li et al. [68] identified eight homogeneous regions in China by applying Ward’s and k-means clustering techniques based on SPI-3, but still encountered subjectivity in the determination of the subregion boundaries attributed to the utilization of station-based data. Drought risk regionalization has only been explored in the research conducted by Wu et al. [69], who took into account the impact of the long-term evolution of drought variables on the results of drought risk regionalization, but only considered the drought severity. In contrast, our study examined the joint probabilities of drought duration and severity at different drought levels, providing more detailed and helpful information for drought risk regionalization. As a result, we divided mainland China into six subregions based on the joint probability. However, it should be noted that the spatial patterns of long-term drought features differed from those on short- and medium-term time scales (seen from Figure 3, Figure 4, Figure 5 and Figure 6) because the time scale played an essential role in the spatial heterogeneity of drought behaviors. Therefore, when discussing drought-related scientific issues, the time scale must be considered according to the research purpose, as pointed out by McKee et al. [6], due to the complexity of drought phenomena.

### 4.3. Limitations and Future Work

This study focused only on a bivariate analysis of drought, specifically duration and severity. However, given that droughts are complex and multifaceted phenomena, relying solely on a bivariate analysis may lead to less accurate estimates of drought probabilities compared to more complex three- or four-variate analyses [70]. Hence, researchers have developed multivariate extensions of bivariate copula that can provide a more comprehensive assessment of droughts [70,71,72,73]. Nonetheless, the parameter estimate and calculation [70,71,72,74] will get more challenging as the number of drought-related variables increases [75]. Additionally, under the backdrop of climate change, the uncertainties surrounding the incidence and progression of drought present an imminent threat to both China’s ecological and food security. Thus, predicting drought in the future becomes crucial. To this end, our forthcoming research will center on employing SPI and high-dimensional copula functions to assess the spatiotemporal evolution of drought, as well as the corresponding drought risk under RCP4.5 and RCP8.5 scenarios. Furthermore, we will conduct a comprehensive analysis of SPI under the two scenarios to explicate the potential uncertainties of drought forecasting.

## 5. Conclusions

Since drought is a serious natural disaster, understanding the characteristics of drought is an essential step in establishing effective measures to alleviate drought-related problems. In this study, China’s monthly gridded precipitation dataset from 1961 to 2020, with a spatial resolution of 0.5° × 0.5°, was used to calculate the SPI values at various time scales and identify drought events in combination with run theory. The features of drought events were described by drought duration and severity, which were fitted by exponential and gamma distributions, respectively. In addition, the Frank copula function was employed to establish the dependence between drought duration and severity. According to the formulas of return period and joint probability, the thresholds of drought duration and severity corresponding to the return periods of 5-, 10-, 20-, 50-, and 100-year under various time scales were calculated, as well as the values of joint probability. As a result, the key findings were as follows:

(1) The spatial patterns of average drought duration and severity indicated that western and northern Xinjiang, western and southern Tibet, western Qinghai, central and northern Yunnan, and eastern Inner Mongolia experienced severe droughts. The spatial patterns of the average drought duration and severity were similar, suggesting that longer drought duration tended to correspond to higher drought severity and vice versa. Although droughts occurred relatively frequently in most of the three northeastern provinces, in the middle and lower reaches of the Yellow River and Yangtze River the severity was relatively mild. Notably, the spatial patterns of the average drought variables on the 12-month time scale differed remarkably from those on the 3- and 6-month time scales.

(2) In the univariate analysis, the spatial coverages of drought duration and severity at various time scales and return periods were also consistent, which further indicated that drought duration and severity were positively correlated. Drought events with longer duration and higher severity mainly occurred in western and northern Xinjiang, southwestern China, and the southern part of the middle and lower reaches of the Yangtze River. Drought events with shorter duration and less intensity were most common in the Greater Khingan Mountains, Changbai Mountains, and Pearl River Basin.

(3) The results obtained from bivariate frequency analysis, using the Frank copula function, revealed that the regions with higher *P* values on the 3- and 6-month time scales were northern Xinjiang, western Qinghai, southwestern China, and the middle and lower reaches of the Yangtze River. Conversely, the regions with lower *P* values were the southeastern coastal regions of China, Changbai Mountains, and Greater Khingan Mountains. On the other hand, for the 12-month time scale, the drought risk was found to be the highest on the Tibetan Plateau.

(4) Mainland China could be divided into six sub-regions according to joint probabilities of drought duration and severity. However, the spatial pattern of drought risk zones exhibited a high degree of similarity on 3- and 6-month time scales but differed from the results on the 12-month time scale.

Overall, regardless of average drought characteristics, joint drought probability, or drought risk regionalization, time scale played an essential role in the spatial heterogeneity of their behaviors, reflecting the complexity of drought phenomena. Additionally, our method, combining SPI and bivariate copulas for drought risk regionalization, is generalizable to other regions and can be applied to other climatic variables, although some modifications may be necessary to fit specific conditions.

## Figures and Tables

**Table 1:** Classifications of drought based on SPI values.

Drought Level	No	Mild	Moderate	Severe	Extreme
SPI value	(−0.5,+∞)	(−1.0,−0.5]	(−1.5,−1.0]	(−2.0,−1.5]	(−∞,−2.0]

## Data Availability

All relevant data are available from the corresponding author upon reasonable request.

## References

[1] Li M., Ge C., Deng Y., Wang G., Chai X. (2020). Meteorological and agricultural drought characteristics and their relationship across the Loess Plateau. Sci. Geog. Sin..

[2] Zhang Q., Yao Y., Li Y., Luo Z., Zhang C., Li D., Wang R., Wang J., Chen T., Xiao G. (2015). Research progress and prospect on the monitoring and early warning and mitigation technology of meteorological drought disaster in Northwest China. Adv. Earth Sci..

[3] Dai A. (2011). Drought under global warming: A review. Wiley Interdiscip. Rev. Clim. Chang..

[4] Spinoni J., Naumann G., Carrao H., Barbosa P., Vogt J. (2014). World drought frequency, duration, and severity for 1951–2010. Int. J. Climatol..

[5] Palmer W.C. (1965). Meteorological Drought.

[6] McKee T.B., Doesken N.J., Kleist J. The relationship of drought frequency and duration to time scales. Proceedings of the 8th Conference on Applied Climatology.

[7] Vicente-Serrano S.M., Beguería S., López-Moreno J.I. (2010). A multiscalar drought index sensitive to global warming: The standardized precipitation evapotranspiration index. J. Clim..

[8] Liu C., Zhang Q., Singh V.P., Cui Y. (2011). Copula-based evaluations of drought variations in Guangdong, South China. Nat. Hazards.

[9] Wang X., Hu C., Wei W., Yu Y. (2016). Temporal and spatial characteristics of drought based on standardized precipitation index in Weibei Loess Plateau. Ecol. Environ. Sci..

[10] Musonda B., Jing Y., Iyakaremye V., Ojara M. (2020). Analysis of long-term variations of drought characteristics using standardized precipitation index over Zambia. Atmosphere.

[11] Zhang Y., Li Z. (2020). Uncertainty analysis of standardized precipitation index due to the effects of probability distributions and parameter errors. Front. Earth Sci..

[12] Wang W., Zhu Y., Xu R., Liu J. (2015). Drought severity change in China during 1961–2012 indicated by SPI and SPEI. Nat. Hazards.

[13] Hayes M., Svoboda M., Wall N., Widhalm M. (2011). The Lincoln declaration on drought indices: Universal meteorological drought index recommended. B Am. Meteorol. Soc..

[14] Svoboda M., Hayes M., Wood D.A. (2012). Standardized Precipitation Index: User Guide.

[15] Yevjevich V.M. (1967). An Objective Approach to Definitions and Investigations of Continental Hydrologic Droughts.

[16] Liu X., Wang S., Zhou Y., Wang F., Yang G., Liu W. (2016). Spatial analysis of meteorological drought return periods in China using copulas. Nat. Hazards.

[17] Li M., Hu W., Wang G., Chai X., Zhang L. (2019). Drought risk in monsoon area of the eastern China based on copula function. Sci. Geog. Sin..

[18] Janga Reddy M., Ganguli P. (2012). Application of copulas for derivation of drought severity–duration–frequency curves. Hydrol. Process..

[19] Kim T.-W., Valdés J.B., Yoo C. (2003). Nonparametric approach for estimating return periods of droughts in arid regions. J. Hydrol. Eng..

[20] Bonaccorso B., Cancelliere A., Rossi G. (2003). An analytical formulation of return period of drought severity. Stoch. Environ. Res. Risk A.

[21] Kim T.-W., Valdés J.B., Yoo C. (2006). Nonparametric approach for bivariate drought characterization using Palmer drought index. J. Hydrol. Eng..

[22] González J., Valdés J.B. (2003). Bivariate drought recurrence analysis using tree ring reconstructions. J. Hydrol. Eng..

[23] Shiau J.T., Modarres R. (2009). Copula-based drought severity-duration-frequency analysis in Iran. Meteorol. Appl..

[24] Xiao M., Zhang Q., Chen X. (2012). Spatial-temporal patterns of drought risk across the Pearl River Basin. Acta Geogr. Sin..

[25] Zhang D., Yan D., Lu F., Wang Y., Feng J. (2015). Copula-based risk assessment of drought in Yunnan province, China. Nat. Hazards.

[26] Shiau J.T. (2006). Fitting drought duration and severity with two-dimensional copulas. Water Resour. Manag..

[27] Masud M., Khaliq M., Wheater H. (2015). Analysis of meteorological droughts for the Saskatchewan River Basin using univariate and bivariate approaches. J. Hydrol..

[28] Zhou B., Xu Y., Wu J., Dong S., Shi Y. (2016). Changes in temperature and precipitation extreme indices over China: Analysis of a high-resolution grid dataset. Int. J. Climatol..

[29] Han L., Zhang Q., Jia J., Wang Y., Huang T. (2019). Drought severity, frequency, duration and regional differences in China. J. Des. Res..

[30] Li M., Ge C., Zong S., Wang G. (2022). Drought assessment on vegetation in the Loess Plateau using a phenology-based vegetation condition index. Remote Sens..

[31] Wang A., Lettenmaier D.P., Sheffield J. (2011). Soil moisture drought in China, 1950–2006. J. Clim..

[32] Zhao C., Deng X., Yuan Y., Yan H., Liang H. (2013). Prediction of drought risk based on the WRF model in Yunnan province of China. Adv. Meteorol..

[33] Han L., Zhang Q., Zhang Z., Jia J., Wang Y., Huang T., Cheng Y. (2021). Drought area, intensity and frequency changes in China under climate warming, 1961–2014. J. Arid Environ..

[34] Lu E., Cai W., Jiang Z., Zhang Q., Zhang C., Higgins R.W., Halpert M.S. (2014). The day-to-day monitoring of the 2011 severe drought in China. Clim. Dyn..

[35] China Meteorological Data Service Centre Dataset of Gridded Monthly Precipitation in China (Version 2.0). http://www.data.cma.cn.

[36] Han Z., Huang S., Huang Q., Leng G., Liu Y., Bai Q., He P., Liang H., Shi W. (2021). GRACE-based high-resolution propagation threshold from meteorological to groundwater drought. Agric. For. Meteorol..

[37] Hong Y., Nix H.A., Hutchinson M.F., Booth T.H. (2005). Spatial interpolation of monthly mean climate data for China. Int. J. Climatol..

[38] Zhao Y., Zhu J., Xu Y. (2014). Establishment and assessment of the grid precipitation datasets in China for recent 50 years. J. Meteorol. Sci..

[39] Shang S., Zhu G., Li R., Xu J., Gu J., Chen H., Liu X., Han T. (2020). Decadal change in summer precipitation over the east of Northwest China and its associations with atmospheric circulations and sea surface temperatures. Int. J. Climatol..

[40] Li Z., Brissette F., Chen J. (2014). Assessing the applicability of six precipitation probability distribution models on the Loess Plateau of China. Int. J. Climatol..

[41] Tang X., Miao C., Xi Y., Duan Q., Lei X., Li H. (2018). Analysis of precipitation characteristics on the Loess Plateau between 1965 and 2014, based on high-density gauge observations. Atmos. Res..

[42] Asadi Zarch M.A., Sivakumar B., Sharma A. (2015). Droughts in a warming climate: A global assessment of standardized precipitation index (SPI) and reconnaissance drought index (RDI). J. Hydrol..

[43] Gumus V., Algin H.M. (2017). Meteorological and hydrological drought analysis of the Seyhan−Ceyhan River Basins, Turkey. Meteorol. Appl..

[44] Paulo A.A., Pereira L.S. (2006). Drought concepts and characterization. Water Int..

[45] Mahmoudi P., Ghaemi A., Rigi A., Amir Jahanshahi S.M. (2021). Recommendations for modifying the standardized precipitation index (SPI) for drought monitoring in arid and semi-arid regions. Water Resour. Manag..

[46] Zhang C., Liu H., Song Y., Liao Y., Duan J., Cai W., Wang S. (2017). Meteorological Drought Grade (GB/T20481-2017).

[47] Chen L., Guo S., Yan B., Li T. (2012). Drought characteristics analysis using copulas. J. Water Resour. Res..

[48] Li M., Zhang Y., Zhang L. (2017). Analysis on drought characteristics of Changchun city in 106 years based on copula function. J. Arid Land Resour. Environ..

[49] Lin H., Wang J., Li F., Xie Y., Jiang C., Sun L. (2020). Drought trends and the extreme drought frequency and characteristics under climate change based on SPI and HI in the upper and middle reaches of the Huai River Basin, China. Water.

[50] Sklar A. (1959). Fonctions de Répartition àn Dimensionset Leurs Marges. Publ. De L’institut De Stat. De L’Université De Paris.

[51] Avsaroglu Y., Gumus V. (2022). Assessment of hydrological drought return periods with bivariate copulas in the Tigris river basin, Turkey. Meteorol. Atmos. Phys..

[52] Akaike H. (1974). A new look at the statistical model identification. IEEE Trans. Autom. Control.

[53] Shiau J.T., Feng S., Nadarajah S. (2007). Assessment of hydrological droughts for the Yellow River, China, using copulas. Hydrol. Process..

[54] Mesbahzadeh T., Mirakbari M., Mohseni Saravi M., Soleimani Sardoo F., Miglietta M.M. (2020). Meteorological drought analysis using copula theory and drought indicators under climate change scenarios (RCP). Meteorol. Appl..

[55] Kendall M.G. (1938). A new measure of rank correlation. Biometrika.

[56] Zuo D., Hou W., Yan P., Feng T. (2014). Research on drought in Southwest China based on the theory of run and two-dimensional joint distribution theory. Acta Phys. Sin..

[57] Shiau J.T., Shen H.W. (2001). Recurrence analysis of hydrologic droughts of differing severity. J. Water Resour. Plan. Manag..

[58] Badr H.S., Zaitchik B.F., Dezfuli A.K. (2015). A tool for hierarchical climate regionalization. Earth Sci. Inf..

[59] Yang P., Xia J., Zhang Y., Zhan C., Qiao Y. (2018). Comprehensive assessment of drought risk in the arid region of Northwest China based on the global palmer drought severity index gridded data. Sci. Total Environ..

[60] Chen Y., Li W., Deng H., Fang G., Li Z. (2016). Changes in Central Asia’s water tower: Past, present and future. Sci. Rep..

[61] Liu Z., Menzel L., Dong C., Fang R. (2016). Temporal dynamics and spatial patterns of drought and the relation to ENSO: A case study in Northwest China. Int. J. Climatol..

[62] Feng W., Lu H., Yao T., Yu Q. (2020). Drought characteristics and its elevation dependence in the Qinghai–Tibet Plateau during the last half-century. Sci. Rep..

[63] Zhao R., Wang H., Zhan C., Hu S., Ma M., Dong Y. (2020). Comparative analysis of probability distributions for the standardized precipitation index and drought evolution in China during 1961–2015. Theor. Appl. Climatol..

[64] Ren G., Ren Y., Zhan Y., Sun X., Yanju L., Chen Y., Wang T. (2015). Spatial and temporal patterns of precipitation variability over mainland China: II: Recent trends. Adv. Water Sci..

[65] Xu K., Yang D., Yang H., Li Z., Qin Y., Shen Y. (2015). Spatio-temporal variation of drought in China during 1961–2012: A climatic perspective. J. Hydrol..

[66] He B., Wu J., Lü A., Cui X., Zhou L., Liu M., Zhao L. (2013). Quantitative assessment and spatial characteristic analysis of agricultural drought risk in China. Nat. Hazards.

[67] Yang X., Li D. (2008). Precipitation variation characteristics and arid climate division in China. Arid. Meteorol..

[68] Li X., Zhou W., Chen Y.D. (2015). Assessment of regional drought trend and risk over China: A drought climate division perspective. J. Clim..

[69] Wu Z., Xu H., Li Y., Wen L., Li J., Lu G., Li X. (2018). Climate and drought risk regionalisation in China based on probabilistic aridity and drought index. Sci. Total Environ..

[70] Ayantobo O.O., Li Y., Song S. (2019). Multivariate drought frequency analysis using four-variate symmetric and asymmetric Archimedean Copula functions. Water Resour. Manag..

[71] Tabari H., Willems P. (2022). Trivariate analysis of changes in drought characteristics in the CMIP6 multi-model ensemble at global warming levels of 1.5, 2 and 3 °C. J. Clim..

[72] Hui-Mean F., Yusof F., Yusop Z., Suhaila J. (2019). Trivariate copula in drought analysis: A case study in peninsular Malaysia. Theor. Appl. Climatol..

[73] Xu X., Xu K., Yang D., Li J. (2019). Drought identification and drought frequency analysis based on multiple variables. Adv. Water Sci..

[74] Valis D., Hasilová K., Forbelská M., Pietrucha-Urbanik K. Modelling water distribution network failures and deterioration. Proceedings of the 2017 IEEE International Conference on Industrial Engineering and Engineering Management (IEEM).

[75] Wang L., Zhang X., Wang S., Salahou M.K., Fang Y. (2020). Analysis and application of drought characteristics based on theory of runs and copulas in Yunnan, Southwest China. Int. J. Environ. Res. Public Health.

